# Alzheimer’s in a dish – induced pluripotent stem cell-based disease modeling

**DOI:** 10.1186/s40035-019-0161-0

**Published:** 2019-07-12

**Authors:** Sherida de Leeuw, Christian Tackenberg

**Affiliations:** 1 Institute for Regenerative Medicine, University of Zurich, Schlieren, Switzerland; 2 Neuroscience Center Zurich, University of Zurich and ETH Zurich, Zurich, Switzerland

**Keywords:** Alzheimer’s disease, Induced pluripotent stem cells, iPSC-derived neurons, iPSC-derived astrocytes, iPSC-derived microglia, Disease modeling

## Abstract

**Background:**

Since the discovery of the induced pluripotent stem cell (iPSC) technique more than a decade ago, extensive progress has been made to develop clinically relevant cell culture systems. Alzheimer’s disease (AD) is the most common neurodegenerative disease, accounting for approximately two thirds of all cases of dementia. The massively increasing number of affected individuals explains the major interest of research in this disease as well as the strong need for better understanding of disease mechanisms.

**Main body:**

IPSC-derived neural cells have been widely used to recapitulating key aspects of AD. In this Review we highlight the progress made in studying AD pathophysiology and address the currently available techniques, such as specific differentiation techniques for AD-relevant cell types as well as 2D and 3D cultures. Finally, we critically discuss the key challenges and future directions of this field and how some of the major limitations of the iPSC technique may be overcome.

**Conclusion:**

Stem cell-based disease models have the potential to induce a paradigm shift in biomedical research. In particular, the combination of the iPSC technology with recent advances in gene editing or 3D cell cultures represents a breakthrough for in vitro disease modeling and provides a platform for a better understanding of disease mechanisms in human cells and the discovery of novel therapeutics.

## Background

AD is the most common age-related neurodegenerative disease affecting memory and executive functions up to behavioral and neuropsychiatric changes in late stages. An estimated 40 million people, mostly older than 60 years, have dementia worldwide, and this number is expected to double every 20 years, until at least 2050 [1]. The World Health Organization predicts that by 2040, neurodegenerative disease will overtake cancer to become the second leading cause of death after cardiovascular diseases [2]. Thus, understanding cellular and molecular pathomechanisms of these disorders is of major importance. Current approved AD therapies are only symptomatic, and no treatment exists to fully stop progression or prevent disease onset.

For decades, primary rodent cell cultures represented the gold standard of mammalian in vitro systems. Despite a significant gain of knowledge on basic neural cell biology and disease pathophysiology obtained from these cells, translation to clinical research remains challenging. Cellular functions, morphology or expression of several disease-relevant proteins such as ApoE strongly differs between rodent and human cells (see following chapters). The establishment of the iPSC culture technique represents one of the most important innovations in biomedical research in this century. By reprogramming somatic human cells into an embryonic stem cell-like state, pluripotent cells are generated which can be further differentiated into virtually any somatic cell. The combination with gene editing techniques such as CRISPR/Cas9 further increases the value of this technique. The newest achievements are 3D models or self-organizing organoids which resemble patient-in-a-dish models of organs and can even display key features of organ-specific cytoarchitecture [3, 4].

Here we review the current research on iPSC-based modeling of AD, highlight current advances and discuss future challenges of the iPSC technology.

## Main text

### Alzheimer’s disease pathophysiology

**Fig. 1:**
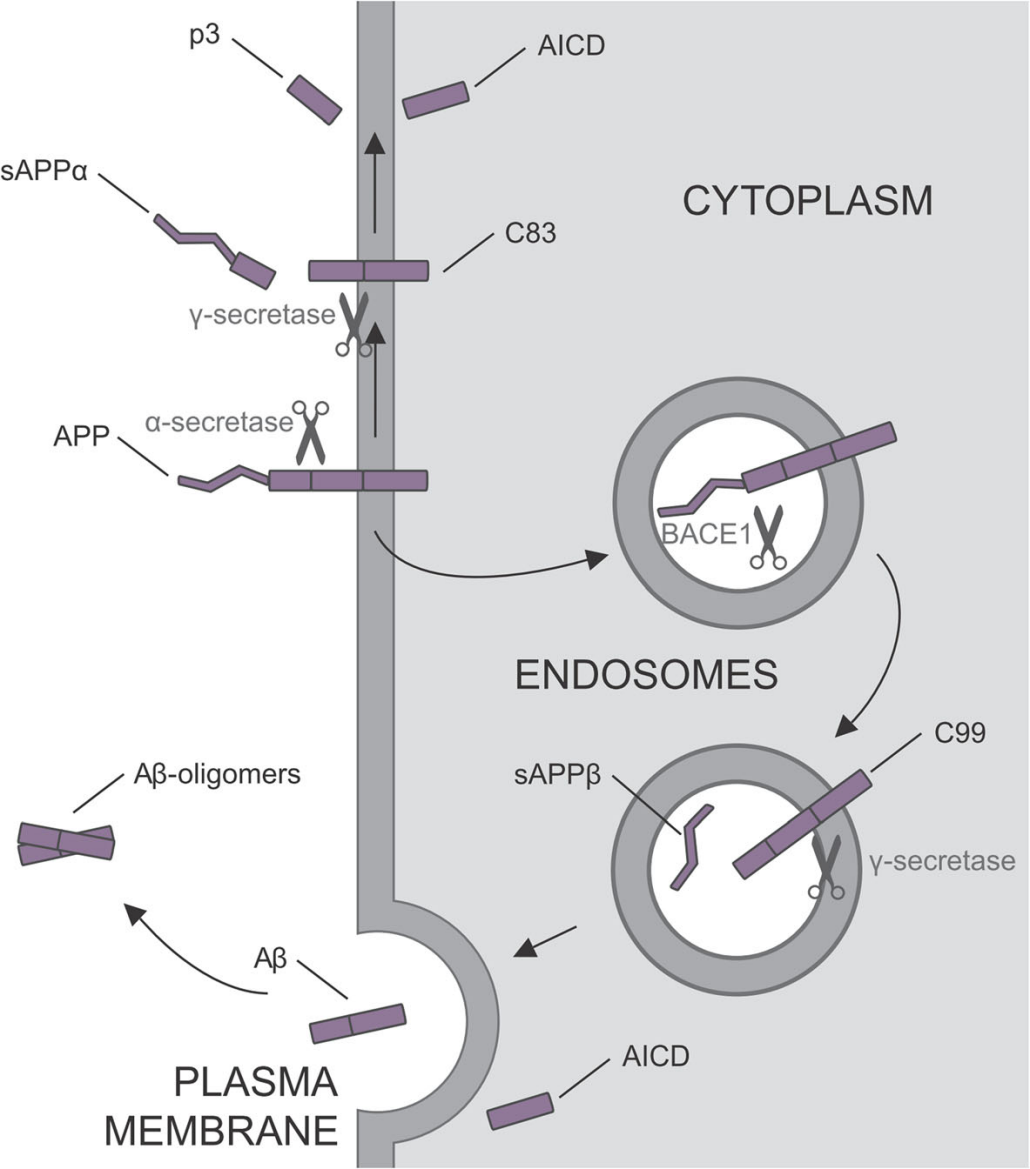
Schematic representation of APP processing

Aggregates of Aβ and hyperphosphorylated tau, i.e. extracellular amyloid plaques and intracellular neurofibrillary tangles are the major hallmarks of AD. The 40-residue peptide Aβ_40_ represents the most abundant Aβ isoform in the brain while the 42-residue Aβ_42_ is the more neurotoxic peptide. Aβ is a processing product of the amyloid precursor protein, APP, which is metabolized by two distinct pathways, the amyloidogenic and non-amyloidogenic pathway. In the latter, which takes place in the endosomal compartment [5], processing of APP by β-secretase (BACE1) generates sAPPβ, the soluble N-terminal ectodomain, and the membrane-bound C99 fragment (Fig. 1). Subsequent cleavage of C99 by γ-secretase causes the production of Aβ which is released from the cell and was originally thought the be the main trigger of AD (also postulated as the “amyloid-hypothesis”) [6], especially in an oligomeric state. Further, APP intracellular domain (AICD) is generated, which can translocate to the nucleus to regulate transcription of approximately 30 genes [7]. In the alternative, non-amyloidogenic pathway α-secretase cleavage at the cell surface generates the C83 fragment and causes the release of sAPPα which has been proposed to be neuroprotective [8, 9]. The α-secretase cleaves APP within the Aβ domain, hence precluding the production of Aβ. However, subsequent processing of C83 by γ-secretase generates a fragment called p3 (Aβ_17-X_). While p3 is not amyloidogenic, it is found in non-congophilic plaques in patients with Down Syndrome and AD, and its relevance for AD pathophysiology is not yet understood [10]. Although most research is conducted on the role of extracellular Aβ, evidence exists that also intracellular deposited Aβ contributes to AD pathology, and that accumulation of intraneuronal Aβ is an early event in the progression of AD, preceding extracellular amyloid plaques [11].

The microtubule-associated protein tau has six isoforms which vary in length due to alternative splicing of exons 2, 3 and 10. Three isoforms contain 3 microtubule-binding repeats (3RTau) the other three isoforms contain 4 repeats (4RTau). In embryonic cells only one of the 3RTau isoforms is expressed, whereas mature neurons show presence of all six isoforms. Tau belongs to the major phosphoproteins in the brain. In the AD brain, tau is abnormally phosphorylated. In addition, tau is subjected to other posttranslational modifications which may further contribute to its pathologic role in AD, such as acetylation or N- and C-terminal truncation (reviewed in [12]). These modifications lead to altered tau folding thereby increasing its aggregation propensity. Aberrantly modified tau mediates toxicity downstream of Aβ [13–15] and knockout of endogenous tau abolished axonal transport deficits in APP transgenic mice [16] confirming its essential role in AD.

Genetically, AD is divided into two forms, familial (fAD) and sporadic AD (sAD). Familial cases have a predominantly early-onset (< 60 years, also called early-onset AD (EOAD)) while sAD cases with no or less familial evidence have later age of onset (> 60 years, late-onset AD (LOAD)) [17]. FAD mutations either 1) increase the total production of Aβ, 2) shift the production to more toxic and aggregation-prone Aβ_42_ or 3) increase the aggregation propensity of the peptide, depending whether the mutation is localized 1) around the β-secretase cleavage, 2) at the γ–secretase cleavage site or 3) within the Aβ sequence [18]. This explains the increased Aβ deposition in the brains of fAD patients. However, fAD accounts for less than 1% of all AD cases [19]. The strongest nongenetic risk factors for sAD are age, medical factors such as diabetes mellitus, high blood pressure or hypercholesterolemia as well as socioeconomic factors such as low education or lack of social contacts [20]. Although sAD is not associated with mutations that ultimately cause disease onset, genetic risk genes play a role in at least 80% of all AD cases [21]. The major genetic risk factor is the presence of the apolipoprotein Ɛ4 (*APOE*4) allele. ApoE is a glycoprotein expressed mainly in the liver and in the brain. It is involved in transport of cholesterol across different tissues and cells and has been implicated in neuronal growth and repair, immune response, and activation of lipolytic enzymes [22]. ApoE has three different isoforms, ApoE2, ApoE3 and ApoE4, which differ only in two amino acid residues. While *APOE*3/3 is the most common genotype and does not affect the AD risk, the presence of one or two copies of *APOE*4 increases the risk by tree or twelve fold, respectively [23]. In vitro, ApoE4 has been shown to enhance aggregation of Aβ compared to ApoE3 [24]. Analyses of ApoE in mouse models show that ApoE4 is associated with increased levels of Aβ, as well as amyloid plaque deposition [25]. Furthermore, the different ApoE isoforms may differentially affect energy metabolism, neuroinflammation and synaptic function [26]. More risk genes have been identified with different population frequencies and odds ratios including TREM2, BIN1, CLU, SORL1, ABCA7, CD33 and others (reviewed in [27, 28]).

Many of the AD risk genes show highest expression in glia, i.e. astrocytes (e.g. *APOE*, *CLU*, *SORL1*) or microglia (e.g. *TREM2*, *CD33*) [28] highlighting the importance of glia, especially microglia and astrocytes, for AD pathophysiology. In our opinion, in the last decade research focused too much on neurons, disregarding the role of glia. However, this recently changed and consequently, the number of studies in iPSC-derived glia increases and new differentiation protocols to obtain human astrocytes and microglia are being developed (see chapter *Differentiation into disease-relevant cell types*). Human glia structurally and functionally differs from murine glia [29–32]. Hence, the use of human iPSC-derived glia is of high importance to i) verify already existing data from animal studies and ii) acquire new insights into the mechanisms by which human glia contribute to the pathogenesis of sAD.

#### iPSCs and disease modeling

**Fig. 2:**
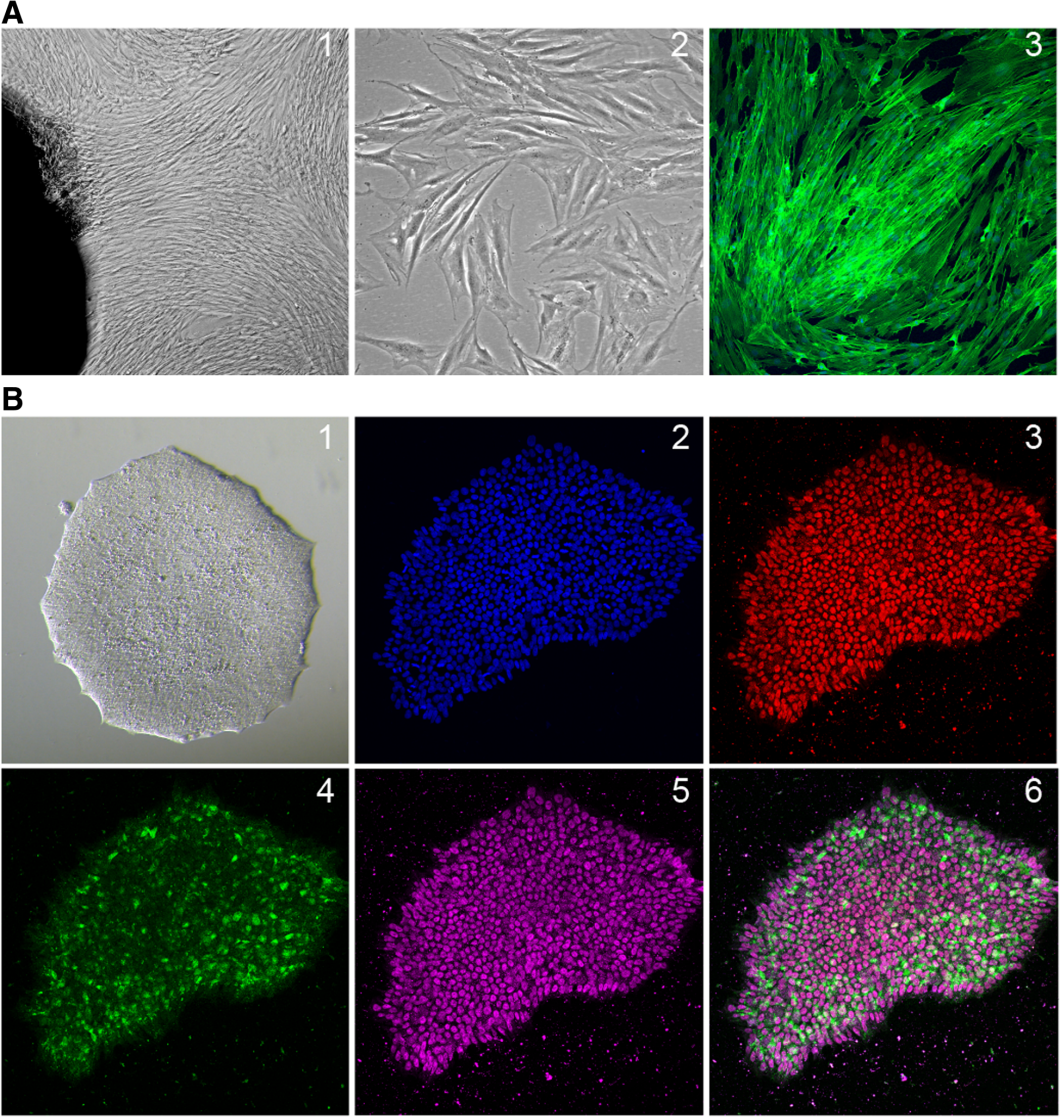
Reprogramming of human fibroblasts (hFib) to iPSCs. **a** Human skin biopsy-derived fibroblasts in culture. [1] hFib growing out of the biopsy (black). [2] Phase contrast image of isolated hFib. [3] hFib stained with Alexa Fluor 488 Phalloidin. **b** IPSC colonies after reprogramming. [1] Light microscopic image of a single iPSC colony. [2–6] Immunostained iPSC colonies showing expression of typical pluripotency marker proteins. [2] DAPI, [3] Oct4, [4] Tra-1-60, [5] Nanog, [6] merged images

Stem cells are defined by the ability to undergo self-renewal, while maintaining an undifferentiated state, and to differentiate into specialized cell types (potency). IPS cells are undifferentiated cells, which have been reprogrammed to an embryonic stem cell (ESC)-like state by expression of defined factors. Thus, many protocols established for ESC culture are applicable for iPSCs as well. However, in contrast to embryonic stem cells, iPSCs can be utilized without concerns of ethical issues or allogeneic immune response when used in regenerative medicine. In 2006 the group of Shinya Yamanaka was the first to generate iPSCs from mouse fibroblasts [33], followed by human fibroblasts in 2007 [34]. Reprogramming was induced by retroviral expression of initial reprogramming factors Oct4 (also called Oct3/4 or Pou5f1), Sox2, Klf4, and c-Myc (together termed OSKM). Over the years, additional factors which increased reprogramming efficiency were discovered, and the combinations of different factors have been refined. Okita and colleagues compared several factor combinations and found that forced expression of RNA-binding protein Lin28, p53shRNA and l-Myc (instead of c-Myc), together with Oct4, Sox2 and Klf4 significantly increased the yield of iPSC colonies after reprogramming human fibroblasts [35]. Creation of a hypoxic environment during reprogramming was found to lead higher numbers of colonies [36]. Further, addition of ascorbic acid, inhibition of GSK3-β [37] or activation of Wnt signaling [38] improved iPSC yield. Moreover, inhibition of histone deacetylases by treatment with sodium butyrate or valproic acid, enhanced the reprogramming process [39, 40]. IPSCs have been generated from many different cell types, including fibroblasts, keratinocytes, cord blood endothelial cells, melanocytes, hepatocytes, T-cells or peripheral blood mononuclear cells, using different combinations of reprogramming factors [41]. Typical iPSC colonies are characterized by a defined edge and expression of pluripotency markers such as Nanog, Oct4 or Tra-1-60 (Fig. 2).

Since the development of the iPSC technology, a major concern included the use of inserting retro viruses into the genome, which may cause inadvertent effects such as tumorigenesis. To circumvent these limitations, non-integrative techniques were established, including episomal vectors [35, 42], Sendai-virus [43], mRNA transfection [44] or piggyback-mediated OSKM transposition [45].

**Fig. 3:**
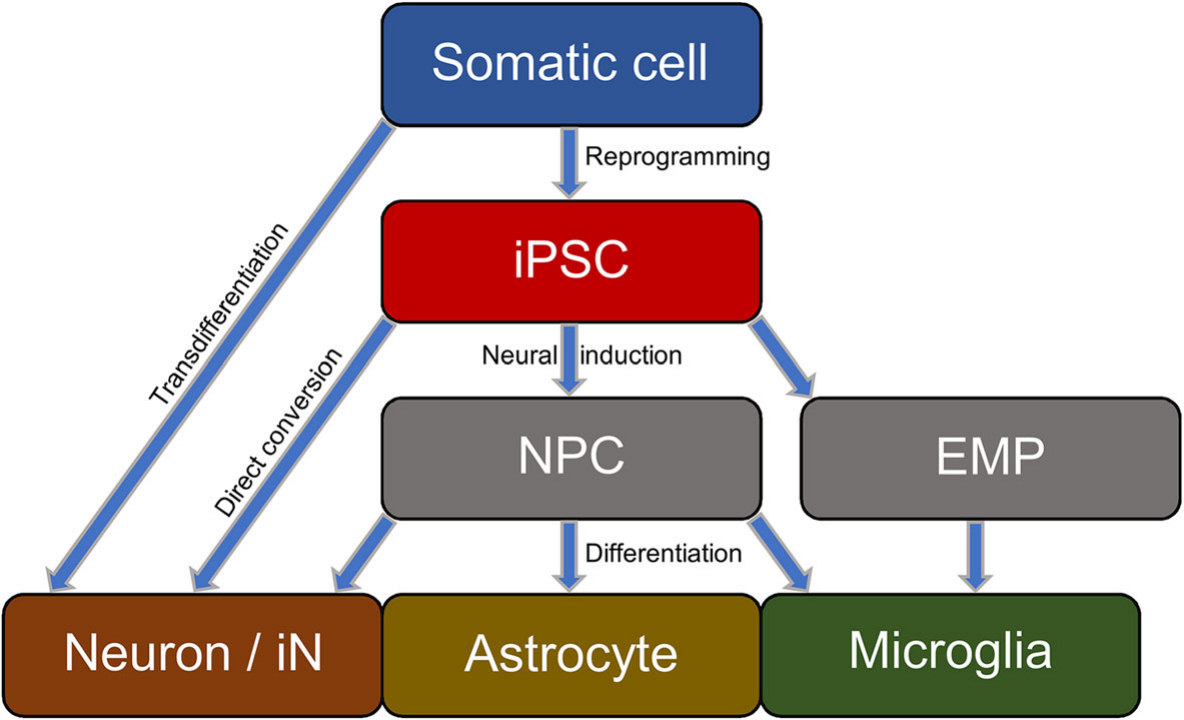
Common ways to generate human neural cells for modeling Alzheimer’s disease. Somatic cells may either be directly converted into induced neuronal (iN) cells (transdifferentiation) or reprogramed into iPSCs. Neural induction – mainly by dual-smad inhibition – yields NPCs, which can further be differentiated into neuron and astrocytes, either pure or of mixed-types. Alternatively, iPSCs can be directly converted to neurons (also called iN cell in this case) by forced expression of transcription factors such as neurogenin2, omitting the NPC step. Microglial cells can be obtained by iPSC differentiation into erythromyeloid progenitors (EMPs) which are further differentiated to microglia. However, few alternative protocols achieve microglial differentiation via NPCs

A further limitation, especially for modeling age-related diseases, is the lack of maturity and aging-signatures of iPSC-derived neural cells. During reprogramming of somatic cells into iPSCs the cellular aging profile is widely erased [46]. Accordingly, neurons differentiated from iPSCs display a fetal, immature phenotype and lack the aging signatures of their source cells [47]. Cellular senescence is characterized by reduction in DNA methylation, heterochromatin, telomere length or nuclear lamina integrity and increase in DNA damage, reactive oxygen species (ROS) formation, or expression of senescence-associated β-galactosidase. Interestingly, fibroblasts from patients suffering from Hutchinson Gildford progeria syndrome, a disease of premature ageing, show these increased senescence signs [48]. HGPS is caused by mutations in the gene for nuclear envelope protein lamin A, resulting in the production of shorter transcript, called progerin. Accordingly, expression of progerin in iPSC-derived neurons induced multiple aging-related characteristics and may facilitate the analysis of late-onset disease features in iPS-derived cells [47]. Another study found the nuclear transport receptor RAN binding protein 17 (RanBP17) to be a master regulator of aging in fibroblasts and iN cells and showed that knock-down of RanBP17 induced cellular aging by disruption of nucleocytoplasmic compartmentalization [49]. A way to circumvent cell rejuvenation during iPSC reprogramming is the direct conversion (transdifferentiation) of fibroblasts into neurons (Fig. 3). This can be achieved by overexpression of defined transcription factors [49, 50] or microRNA/transcription factor combinations [51, 52]. These directly converted neurons, called induced neuronal cells (iN cells), retain their aging profile and displayed age-related disease phenotypes when taken from Huntington’s or Parkinson’s patients [47, 53].

**Fig. 4:**
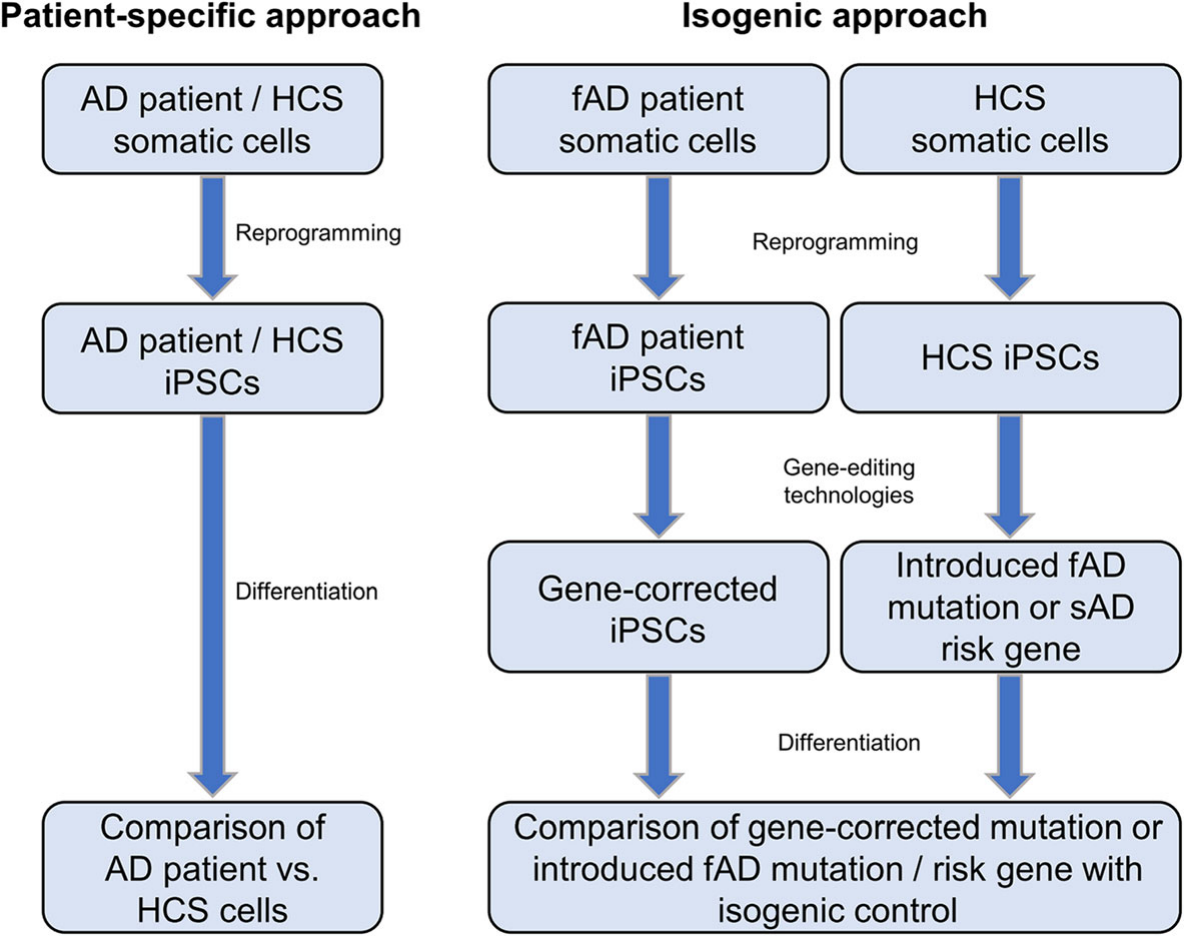
Different approaches of creating human AD cell models. The classical patient-specific approach starts with somatic cells from sAD or fAD patients and healthy control subjects. After reprogramming and differentiation cultures of patient-derived cells are compared to HCS-derived cells. In the monogenic / isogenic approach somatic cells are taken from either fAD patients or healthy controls. Using gene-editing technologies the fAD mutation is corrected or a fAD mutation is introduced into HCS cells. Alternatively, a sAD risk gene can be introduced or modified in HCS cells, such as APOE. After differentiation of into the desired cell type, these modified cells are compered to their isogenic controls, reducing interpatient variation

Nevertheless, the iPSC approach has several advantages over transdifferentiated cells as it permits obtaining large numbers of cells which can be kept in culture theoretically indefinitely and allows gene-editing at this stage. Gene editing technologies further increase the power of iPSCs for both, disease-modeling and regenerative therapies. IPSC-based disease modeling can principally be achieved by two different approaches, a patient-specific and an isogenic approach (Fig. 4). Several studies generated iPSCs from sAD or fAD patients and determined AD-related phenotypes compared to cells from healthy control subjects (see chapter *AD pathophysiology in iPSC-derived cells* for more details). While this approach is a first and important step to gain insights into AD pathomechanisms, the interpretation of the obtained data must be taken with care. Due to high genetic variability between individuals, the observed finding cannot be linked to the disease progression of the respective patient with full certainty. As gene-editing technologies become more and more easy applicable and cell banks arise providing large stocks of gene-edited iPS cell lines, we recommend using the isogenic approach wherever possible. In this approach genome-editing such as CRISPR/Cas9 is used to either correct a mutation in cells from a fAD patient or to introduce a mutation in cells from HCSs. These cells are then compared to their parental (isogenic) line thereby eliminating bias caused by interpatient genetic variation. This approach can even be used to model sAD, for example by introducing/mutating AD risk genes such as *APOE*.

#### Differentiation into disease-relevant cell types

##### Neuronal cells

Pluripotent cells have the capacity to differentiate into three germ layers: endoderm, ectoderm and mesoderm. In turn, these multipotent cells can give rise to terminally differentiated cell types. Ectoderm gives rise to neuroectoderm and NPCs, which can finally be differentiated into neuronal or glial cells (i.e. astrocytes and oligodendrocytes). During neurodevelopment strict temporal and spatial expression of patterning factors determine cell fate. The most pivotal transcription factors are SMAD transcription factors, which play a fundamental role in transforming growth factor β (TGFβ) and bone morphogenic protein (BMP) signaling [54]. In these pathways two different classes of receptor-regulated SMAD transcription factors, SMAD1/5/8 and SMAD2/3, are responsible for the respective signaling pathway. Inhibition of both classes of SMAD transcription factors is in turn a defining part of neuralation as their inhibition leads to the formation of the neural tube [55]. During indirect neuronal differentiation, antagonizing TGFβ and BMP receptors causes dual SMAD inhibition, and has been shown effective in iPSC differentiation to neural cells [55], yielding over 80% of PAX6 positive neural progenitor cells (NPCs). Currently, NPC yields have been improved by the use of more stable, specific, and efficient small molecules [56]. NPCs in turn can be differentiated to a mixed population of neuronal cells. Specific morphogens can be applied directing a specific cell fate. Sonic hedgehog induces a ventral fate such as GABAergic and motor neurons or sonic hedgehog in combination with retinoic acid induces midbrain dopaminergic neurons [55, 57–59].

To overcome laborious methods and impure yields, direct conversion of iPSCs to neurons was developed [60, 61] (Fig. 3). Viral overexpression of Ngn2 yielded a pure population of excitatory glutamatergic neurons within 2 weeks, called iN cells [62]. Besides the quick and efficient aspects of directed differentiation, it also allows for a pure single cell type culture. This can be an advantage in order to study cell type-specific phenotypes, which can be diluted out in mixed neuronal cultures, and therefore not detected. In the scope of AD research, this enables the definition of pathogenic contributions of distinct cell types.

##### Astrocytes

Until recently, astrocyte differentiation protocols were laborious and inefficient [63, 64]. By applying astrocyte-specific morphogens to NPCs, such as fibroblast growth factor 2, Heregulin-1β, insulin-like growth factor 1 and Activin A, functional astrocytes can now be derived within 30 days from the NPC stage [65]. Alternatively, astrocytes can be differentiated from glial progenitor cells (GPCs). GPCs are generated similar to NPCs, however, platelet-derived growth factor was added during progenitor development. Addition of N2, B27, serum and Leukemia inhibitory factor to the GPCs resulted in formation of astrocytes after a total culture time of 6 to 8 weeks [66]. Similar to induced neurons, it is also possible to directly convert fibroblasts to astrocytes by the forced overexpression of *NFIA*, *NFIB* and *SOX9*, yielding functional astrocytes in 2 weeks [67]. However, this was only shown for murine fibroblasts. Human fibroblasts could be directly converted to astrocyte-like precursors by applying a chemical cocktail (histone deacetylase inhibitor valproic acid, the GSK3β inhibitor CHIR99021, the TGFβ inhibitor 616,452, the lysine specific histone demethylase LSD1 inhibitor tranylcypromine, the cyclic AMP inducer forskolin, and a histone methylation inhibitor DZNep). Astrocyte-like precursors had to be further differentiated into astrocytes but the efficiency was rather low with 15% GFAP positive and 40% S100β positive cells [68].

Simplified protocols repurposed commercial primary astrocyte maintenance medium for astrocyte induction when applied to NPCs [69]. Both types of methods yielded functionally active astrocytes, which were able to take up glutamate and showed an immune response to several stimuli [65, 66, 69], including Aβ peptides, making these cells especially suitable to elucidate pathomechanisms in AD. This was evident by the switch from supportive glycolytic astrocytes, to oxidative, reactive astrocytes in cells expressing a PS1 fAD mutation [70], as well as a lack of neuronal support shown in sAD iPSC-astrocyte models [71, 72]. This supports the general understanding that astrocytes in AD lose their homeostatic support function and gain a toxic effect, thereby actively contribute to neuronal dysfunction in AD.

##### Microglia

The development of microglia does not have an ectodermal origin, in fact, this cell type is derived from mesodermal hematopoietic stem cells, specifically yolk sack-derived c-Myb-independent primitive macrophages [73]. It was not until recently that feasible microglia differentiation protocols were developed (see [74] for a complete overview). In short, recent studies pushed the development of erythromyeloid progenitors (EMPs), which are then directed to myelopoiesis by application of morphogens such as interleukin 3, and macrophage colony stimulating factor (M-CSF), additional factors are optional [75, 76], with one study using an initial selection step [77]. Further, maturation and microglial enrichment requires supplementation with CSFR1 ligand interleukin 34 instead of M-CSF [78]. IPSC-microglia were able to phagocytose fibrilar Aβ [75], express AD relevant genes such as *APOE* and *TREM2* [75, 78], and were reactive in co-culture with neurons expressing fAD mutations [79]. Furthermore, these genes were upregulated when microglia were treated with fibrilar Aβ [75]. In addition, studies focusing on other neurodegenerative disorders have found specific phagocytosis deficits in iPSC-microglia, a mechanism also known to be affected in AD [80] indicating that iPSC-derived microglia could be an appropriate cell model to study AD pathogenesis.

##### 3-dimensional models

Neural cells are routinely differentiated in a conventional two-dimensional (2D) culture setting. However, differentiating iPSCs in 3D cultures has been shown to increase cellular maturation, evident by elevated expression of synaptic marker synaptotagmin and neural cell adhesion molecule 1 in NPC-derived neurons [81]. It should be noted that these NPCs, named ReN cells, did not originate from iPSCs. ReN cells are immortalized human neural progenitor cells, originally derived from the ventral mesencephalon region of the human fetal brain, which have the ability to differentiate into neurons and glial cells. 3D cultures can be established through culturing cells in scaffolds, such as Matrigel or synthetic hydrogels, or scaffold-free by self-organizing or transcription factor patterned organoids [3, 60, 82–84]. Using single type cell cultures [60], the effect of 3D culture conditions can be easily analysed for a certain cell type while the microenvironment, especially in hydrogel-based 3D cultures, is controllable. However, single cells do not recapitulate the complexity of neurons interacting with each other and with glial cells. On the other hand, the microenvironment and access to nutrients in self-organizing organoids cannot be controlled well, in addition to lack of cytoarchitectural structures. Patterned 3D corticospheres allow for the development of structured cortical lamination comprising of both superficial and deep cortical layer neurons, including non-reactive astrocytes [84]. Allowing the spheres to grow in suspension rather than on semi-solid Matrigel or hydrogel enables the access of transcription factors and thus closer control of the cell microenvironment. However, to achieve matured 3D organoids the center will be deprived of nutrients and oxygen, creating a necrotic core, which may confound meaningful results [3]. To overcome this, vasculature will need to be integrated into the organoids, which has been tried, but is still in a preliminary stage when using iPSCs (see [85] for more comprehensive review). While all 3D model systems have their advantages and disadvantages, evidently, these models showed valuable improvements to understanding AD pathology as mentioned below.

Even though iPSC-derived neural cells can mimic AD pathomechanisms to a certain extent in 2D cultures, the presence of robust Aβ plaque and neurofibrillary tangle pathology is overtly absent [81]. By using 3D cultures, Aβ peptides are trapped in the matrix and not removed during medium changes and can therefore form aggregates in the 3D environment. Evidently, fAD models showed increase Aβ aggregations in hydrogel-based neuro-organoids [3] and single cell models [81]. Another major finding was that Matrigel-based 3D cell cultures expressed 4RTau isoforms, which were not detected in 2D cultures [81] at the same time point. In long-term cultures (~ 6 months) of dopaminergic neurons, 4RTau expression was observed also in 2D [86]. In addition to Aβ and Tau pathology, other AD-related pathways such as P21-activated kinase (PAK) were also found to be dysregulated in fAD cells in 3D but not in 2D [60]. PAK signalling is sensitive to mechanotransduction, which may explain why this pathway is affected by fAD mutations in 3D cultures, but is due to its absence not affected in 2D cultures. When exposed to Aβ aggregates a significant PAK-mediated response was observed, which was absent in 2D cultures [60]. Using 3D iPSC-neuronal models could allow for the study of AD relevant pathomechanisms in a setting that more closely reflects the in vivo context.

#### AD pathophysiology in iPSC-derived cells

##### Aβ and tau pathology

A prominent pathological feature of AD, the increased production of Aβ_42_, Aβ_40_ and increased Aβ_42/40_ ratio, was replicated in iPSC-derived mixed neuronal cultures generated from fAD fibroblasts [63, 87–89] and also in fAD NPCs [90]. However, Aβ pathology was shown to be cell type specific, as fAD and sAD fibroblasts did not secrete increased levels of Aβ_40_ [89]. Not only neurons but also fAD astrocytes were shown to secrete increased levels of Aβ_42_ and displayed reduced capacity to take up Aβ_42_ [70, 91], evidently showing that astrocytes are not mere bystanders in AD pathology. Besides aberrant Aβ metabolism, AD is also defined by the presence of hyperphosphorylated Tau aggregates. As described above, iPSC-derived neurons are known to be resemble immature neurons and do not express 4RTau in the timeframe of in vitro neuronal differentiation [92]. 4RTau isoforms are more prone to aggregate due to the additional microtubule-binding domain. When iPSC-neurons were cultured for an extended period of time, up to 356 days in vitro*,* a shift from solely 3RTau expression to co-expression with 4RTau was observed [93].

Even in the absence of 4RTau expression, iPSC-neurons from both fAD and sAD patients showed an increase in phosphorylated tau (pTau) [89, 94]. In fAD neurons carrying an APP duplication (APP^dp^), major Tau kinase GSK-3β showed increased activity which was rescued by β-secretase inhibition [89]. In addition, extracellular application of Aβ to HCS-derived iPSC-neurons induced Tau phosphorylation without affecting total Tau protein levels [95].

Even though most hallmarks were prominent in mixed neuronal cells derived from fAD patients, sAD patient-derived neuronal cells did not show a robust Aβ_42/40_ ratio or pTau increase [63, 89, 96, 97]. An explanation for these irregular observations in sAD neurons is the extensive variation of genetic background as described above. In addition, the heterogeneity of these cultures could mask distinct pathomechanisms in specific cell types. Using pure cultures of distinct neuronal subtypes could indicate which cell type contributes to specific disease mechanisms in sAD [89, 98].

##### APP cleavage

Elevated Aβ production in neuronal cells is caused by aberrant APP processing. In iPSC-neurons the APPV717F mutation caused increased generation of Aβ and sAPPβ [87], induced by mislocalization and enrichment of APP in early endosomes. As BACE-1 shows highest activity in early endosomes, the accumulation of APP in its close proximity enables BACE-1 to readily cleave an abundance of APP. Enlarged endosomes have been observed in APP^dp^ neurons but not in fAD neurons carrying the PSEN1ΔE9 mutation [89, 99]. Nonetheless, in the absence of enlarged or increased numbers of endosomes, a decrease in APP endo- and transcytosis was found [100]. In addition, elevated levels of Rab11 were observed - a molecule playing a prominent role in receptor recycling and transport. Endocytosis of low-density lipoprotein (LDL) was also shown to be defective, with the decrease of LRP1 surface expression suggested as causative mechanism. These endocytosis deficits were rescued with β-secretase inhibition and were not dependent on the specific fAD mutation (e.g. APP or presenilin 1 (PS1)) [100]. Consistently, sAD neuronal models also displayed deficits in endocytosis, for example by decrease of the endocytosis-mediating protein clathrin [97]. The presence of endosome pathology independent of AD type or presence of APP or PS1 mutations, indicate that endocytosis and vesicle recycling may be a fundamental factor in AD pathogenesis.

##### APOE

As the presence of the *APOE*4 allele is the major genetic risk factor in sAD, this field of research has recently refocussed on neural models expressing different *APOE* variants, using either isogenic or patient derived cells distinguished based on *APOE* status [71, 72, 101]. ApoE is mainly expressed by astrocytes in the brain, and only expressed by neurons under stress conditions or trauma [102]. Patient-derived iPSC-astrocyte models carrying homozygous *APOE*3 or *APOE*4 secreted ApoE with a different lipidation status, apoE4 lipoprotein particles were less lipidated compared to  ApoE3 particles [72]. Furthermore, isogenic homozygous *APOE*4 astrocytes both produced and secreted less ApoE than *APOE*3/3 cells [71]. Gene regulation in *APOE*4/4 astrocytes was different compared to *APOE*3/3 astrocytes, with genes associated with lipid metabolism being prominently affected. Additionally, *APOE*4/4 astrocytes were less efficient in clearing Aβ42 which was shown to be a lysosome dependent effect [71]. *APOE*4/4 astrocytes had a decreased ability to support neuronal and synaptic homeostasis, as neurons in co-culture expressed decreased amounts of neuronal and synaptic markers, in addition to decreased survival rates shown for isogenic [71], and patient-derived iPSC-astrocytes [72].

Neurons carrying the *APOE*4/4 allele showed increased Aβ_40_, Aβ_42_ and sAPPβ secretion as well as elevated Tau phosphorylation [71, 101]. Interestingly, inhibition of Aβ production did not decrease pTau in these cells. In a mixed neuronal culture expressing ApoE4, especially GABAergic neurons showed prominent degeneration due to increased apoptosis [101]. Defective GABAergic neurons have previously been described in (pre-clinical) AD, and non-demented subjects carrying *APOE*4, which can cause network hyperexcitability [103]. During differentiation, *APOE*4/4 cells take on a neuronal fate earlier than *APOE*3/3 cells and lose their ability to proliferate [71]. Subsequently, *APOE*4 neurons showed an increase in synaptic density and neurotransmitter release, eventhough synaptic genes were downregulated, indicating the presence of an overcompensatory mechanism. These structural alterations could lead to network hyperexcitability, a known phenomenon in AD described above. Corresponding to endosomal pathology found in sAD and fAD neurons described earlier, *APOE*4/4 neurons also displayed an increased number of early endosomes. Genetic correction of *APOE*4/4 to *APOE*3/3 decreased these phenotypes [71, 101]. The *APOE*4/4 effects were also ameliorated by small molecule structure corrector PH002, which changes the conformation of ApoE4, resembling the conformation of ApoE3, as physiological Aβ_40_/Aβ_42_ ratio was restored and pTau levels were decreased [101]. In addition, neuronal cells from a patient lacking the *APOE* gene (*APOE* −/−), showed a phenotype similar to *APOE*3/3 neurons, indicating that ApoE4 could have a gain of toxic function.

Next to astrocytes, microglia are the second major contributor of ApoE, especially plaque-associated ApoE, in the AD brain [104]. IPSC-microglia carrying the *APOE*4/4 genotype showed a positive regulation of immune system-related genes, whereas genes involved in cell movement and development were downregulated [71]. These cells had shorter and fewer processes compared to *APOE*3/3 microglia and showed slower phagocytosis of Aβ_42_. However, when seeded in neuronal organoids, *APOE*4/4 microglia display longer processes. Microglia with shorter processes have been described to be more efficient in Aβ uptake in the brain, indicating *APOE*4/4 microglia in the 3D environment have a reduced ability to sense Aβ_42_, which resulted in an increase in extracellular Aβ levels in the neuro-organoid.

##### Synaptic pathology

Aβ has been described to interfere with synaptic processes and mediate synaptic degeneration and toxicity (reviewed in [105]). Neuronal and astrocytic iPSC-derived cells are electrophysiologically functional evident by spontaneous and evoked activity [89, 95, 106]. In addition, pre- and post-synaptic markers have been detected within close proximity, indicating the presence of functional synapses. Hence, iPSC-derived models enable the study of synaptic function and dysfunction. Synaptotoxic processes previously established in other AD models have likewise been observed in iPSC-derived neurons. Application of Aβ to healthy iPSC-neurons reduced the number of synaptic recycling pool (RP) vesicles but did not affect synaptic vesicle release or the abundance of pre-synaptic puncta [95]. The RP vesicle deficit was caused by a defective synaptic vesicle reuptake mechanism, whereas the exocytosis kinetics were unchanged (e.g. no change in synaptic vesicle release). Both, exogenous Aβ application and endogenous Aβ in fAD neurons failed to show pre-synaptic mechanistical effects [89, 95]. However, in the post-synapse deficits were detected in the form of reduced post-synaptic puncta, and reduced AMPA receptor-mediated mEPSCs, indicating that Aβ had a pronounced post-synaptic effect in iPSC neurons [95]. Interestingly, pre- and postsynaptic protein levels were also unaffected in cultures of sAD patient-derived glutamatergic neurons in which Aβ and pTau levels were not dysregulated [98]. This could imply that either Aβ and/or pTau pathology is needed for a synaptotoxic phenotype, or the mere presence of one neuronal cell type (glutametergic neurons) cannot recapitulate the intricacy of a complex in vivo synaptic system.

##### Endoplasmic reticulum and mitochondrial stress

Mitochondrial metabolism, ER homeostasis and lysosomal clearance are essential to maintain neuronal and glial functions. In AD, however, these pivotal organelles have been implicated in contributing to neuronal degeneration. Early pathological events include the accumulation of misfolded protein in the ER, triggering a stress mechanism called the unfolded protein response (UPR) [107]. Unfolded protein can be sequestered and cleared by autophagy, which appears to be dysfunctional in AD. The UPR was upregulated in fAD and sAD iPSC-neurons [63], as well as in healthy iPSC-neurons after application of Aβ, where ER stress proteins LC3-II, BiP and CHOP were increased [95]. The aberrant accumulation of these proteins recapitulated the aforementioned deficits in autophagy. Accordingly, autophagy markers LC3-II and p62 levels where increased in fAD fibroblasts and neurons [108, 109]. In addition, decreased acidification of lysosomes, and increased levels of early endosomes/lysosomes were detected, indicating that the autophagic degradation phase was obstructed and lysosomal functioning was compromised [109]. Further, presenilin-deficient iPSC-neurons showed altered lysosomal function, reduced autophagy and decreased levels of cytosolic and nuclear calcium [108]. Dysfunctional calcium homeostasis was also observed in iPSC-astrocytes derived from a patient with the PSEN1ΔE9 mutation, contributing to loss of homeostatic neuronal support by astrocytes [70].

Oxidative stress, caused by increased mitochondrial ROS production, is a central process in normal aging. However, aberrant ROS production is observed in AD brains and has been linked to disease progression. Alterations in mitochondrial membrane composition, structure and subcellular localization of mitochondria were reported in AD [110]. Accordingly, increased ROS levels were observed in iPSC-derived neurons and astrocytes from fAD and sAD patients [63, 98]. Interestingly, protein levels of mitochondrial oxidative phosphorylation chain complexes were upregulated in sAD neurons in the absence of alterations in Aβ or pTau, indicating that mitochondrial dysfunction may be an early event upstream of Aβ or Tau pathology [98].

## Conclusions

Stem cell-based disease models have the potential to induce a paradigm shift in biomedical research. Although several concerns still limit the value and validity of iPSC-based studies, most of these limitations can be overcome by following some general guidelines. Studies on iPSC-based modeling of AD are often not reproducible or show opposite results. Better standardization of differentiation protocols – currently most labs use their own protocols, better characterization of the obtained cell cultures and use of more defined media and coatings will reduce the variability among labs and increase reproducibility. As described above, another concern, which is related to poor reproducibility, is the heterogeneity among different individuals and the heterogenous nature of sAD. To overcome this limitation, higher donor numbers, patients as well as controls, will be necessary. Alternatively, isogenic cells should be used instead of AD patient-derived cells, wherever possible. This further allows the analysis of specific disease risk genes, such as *APOE* or *TREM2*. To increase cellular maturity of iPSC-derived cells, which is lost during reprogramming of adult somatic cells, 3D cell cultures and organoid technologies represent a major step forward.

Taken together, iPSCs – either patient-derived or isogenic – constitute a breakthrough in modeling human diseases. Although several hurdles still have to be overcome, this technique allows understanding the earliest events in AD and connecting them to their underlying molecular mechanisms, providing the basis for identification of disease pathomechanisms and new therapeutic targets.

## Data Availability

Not applicable.

## Abbreviations

2D: Two-dimensional
3D: Three-dimensional
AD: Alzheimer’ s disease
*APOE*: Apolipoprotein E
APP: Amyloid precursor protein
Aβ: Amyloid-β
Cas: CRISPR-associated protein
CRISPR: Clustered regularly interspaced short palindromic repeats
EMP: Erythromyeloid progenitors
fAD: Familial AD
hFib: Human fibroblasts
HPC: Hematopoietic progenitor cell
IL: Interleukin
iN cell: Induced neuronal cell
iPSC: Induced pluripotent stem cell
NCAM1: Neural cell adhesion molecule 1
NPCs: Neural progenitor cells
PAK: P21-activated kinaseren
PS1: Presenilin 1 protein
PSEN: Presenilin
PSEN1: Presenilin 1 gene
p-Tau: Phosphorylated tau
RanBP17: RAN binding protein 17
sAD: Sporadic AD
